# High Expression of JMJD4 Is a Potential Diagnostic and Prognostic Marker of Renal Cell Carcinoma

**DOI:** 10.1155/2021/9573540

**Published:** 2021-12-24

**Authors:** Hao Yan, Yewei Bao, Zongming Lin

**Affiliations:** ^1^Department of Urology, Zhongshan Hospital, Fudan University, China; ^2^Department of Urology, Changhai Hospital, Naval Medical University, China

## Abstract

Histone demethylase JMJD4 is a burgeoning tumor marker, which has been proven to be associated with colon cancer, but the role it plays in kidney cancer has not yet been investigated. In the present study, we evaluated whether JMJD4 can be a prognostic marker of patients with clear cell renal cell carcinoma (ccRCC) using data from public platform and *in vitro* experiments. Our results revealed that the expression of JMJD4 is higher in cancerous tissue than in normal tissues (*p* < 0.001). High expression of JMJD4 is associated with a poor overall survival (OS) of ccRCC as compared with low expression of JMJD4 (*p* = 0.015). JMJD4 showed significant relevance with M stage (*p* = 0.016), gender (*p* = 0.003), OS (0.018), disease-specific survival (DSS) (0.007), and percussion free interval (PFI) (0.041). Univariate and multivariate Cox analyses demonstrated that high JMJD4 expression had independent predictive value for OS in ccRCC patients (hazard ratio (HR) = 1.563, 95%confidence interval (CI) = 1.055‐2.316, and *p* = 0.026). Besides, *in vitro* experiments confirmed that high expression of JMJD4 can significantly promote the invasion ability (*p* < 0.001), cloning ability (*p* < 0.001), and proliferation (*p* < 0.001) of renal cell carcinoma. In summary, high JMJD4 expression may be a prognostic marker in patients with kidney cancer.

## 1. Introduction

Renal cell carcinoma (RCC) is a common malignant tumor of the genitourinary system. It can be mainly divided into three subtypes, clear cell renal cell carcinoma (ccRCC), chromophobe, and papillary renal cell carcinoma, which comprise 65–70%, 15–20%, and 5–7% of all RCCs [1, 2]. RCC accounts for nearly 3% of all adult malignant tumors, and its incidence and related mortality have been increasing globally in the past decades [3, 4]. Usually, some patients have been prompted to have advanced RCC at the time of diagnosis due to the insidious onset of RCC. This tricky feature leads to poor prognosis and high mortality of patients with RCC. In advanced RCC, increased resistance to targeted therapies which may be mediated by gene disorders, angiogenesis, and decreased intake of tyrosine kinase inhibitors by cancer cells is common. In view of the hidden onset of RCC and the high resistance rate of targeted drug therapy for advanced RCC, it is urgent to explore effective biomolecular markers to contribute to the early diagnosis and prognostic information for RCC.

The modification of histones is one of the epigenetic mechanisms that regulate gene expression without changing the genotype [5, 6]. Hitherto, histone modifications that have been widely studied are histone methylation and acetylation modifications, which are closely related to the occurrence of many cancers [7, 8]. Histone demethylase is one of the most important modification enzymes involved in the regulation of chromatin function by catalyzing the removal of the methyl group on the N-terminal lysine residue of histone [9]. Many histone demethylases have been proven to promote the genesis and development of cancer, such as KDM3A, KDM4A, KDM4D, and JMJD6. Study has confirmed that KDM3A, which is highly expressed in colorectal cancer, is conducive to metastasis of colorectal cancer [10]. KDM3A has also been found to be increased in pancreatic tumors and promote cancer genesis by regulating expression of DCLK1 [11]. KDM4A could function as an oncogene in lung cancer by mediating expression of Myc through the Wnt/*β*-catenin signaling pathway [12]. In our previous study, we found that KDM4D inhibition can reduce the proliferation and development of kidney cancer. In addition, JMJD6 has been confirmed to be related to the progression of kidney cancer recently [13]. JMJD4 is a newly discovered histone demethylase which is homologous to JMJD6. However, the relationship between JMJD4 and tumor, especially kidney cancer, has not been reported yet.

This study is aimed at evaluating the prognostic value of JMJD4 in kidney cancer. We also conducted *in vitro* experiments to evaluate the effect of JMJD4 on ccRCC cells. Our results demonstrated that JMJD4 may be a cancer-promoting gene, and high expression of JMJD4 portends a poor prognosis in these patients.

## 2. Materials and Methods

### 2.1. Data Acquisition

The gene expression data and clinical data of ccRCC patients (Workflow Type: FPKM) were acquired from the online TCGA website (https://portal.gdc.cancer.gov/). Immunohistochemistry staining of ccRCC tissues was downloaded from The Human Protein Atlas (https://www.proteinatlas.org/). Patients with missing data, unknown staging status, and unknown survival status were excluded. Finally, a total of 539 patients with ccRCC with clinical characteristics information were obtained.

### 2.2. Gene Ontology (GO) and Kyoto Encyclopedia of Genes and Genomes Pathway (KEGG) Enrichment Analysis

Differentially expressed genes in the low and high JMJD4 data set were identified and categorized into specific signaling pathways by GO (https://www.geneontology.org) and the KEGG database (http://www.genome.ad.jp/kegg/). The significant GO and KEGG pathways were identified by Fisher's exact test. Adjusted *p* value < 0.05 and *q* value < 0.2 were considered as statistically significant.

### 2.3. Cell Culture

Human kidney cancer cell line (Caki-1), purchased from American Tissue Culture Collection (ATCC; Manassas, VA, USA), were cultured in McCoy's 5A (HyClone, Logan, UT, USA) medium at 37°C, 5% CO_2_ in a humidified environment. All media are supplemented with 10% fetal bovine serum (Hyclone). Then, cells were transfected with pcDNA-3.1(+)-JMJD4 plasmid and control vector plasmid.

### 2.4. Quantitative Real-Time Polymerase Chain Reaction (qRT-PCR) Analysis

Caki-1 cells were harvested, and total RNA was extracted. Then, cDNA was synthesized by EntiLink™ 1st Strand cDNA Synthesis Kit (ELK Biotechnology, Wuhan, China) according to the manufacturer's instruction. Quantitative real-time PCR was performed using the StepOne™ Real-Time PCR (Life technologies, Wuhan, China) with the following primer sequences: JMJD4 sense primer, 5′-AGATGGTGTTTGTGCCCAGT-3′; JMJD4 antisense primer, 5′-CATGTTGGCCAGGTTGAAGC-3′; *β*-actin sense primer, 5′-CATCATCCCTGCCTCTACTGG-3′; and *β*-actin antisense primer, 5′-GTGGGTGTCGCTGTTGAAGTC-3′.

### 2.5. Colony Formation Assay

Caki-1 cells were digested and resuspended in medium and seeded on a 6-well plate at a density of 500 cells per well. The culture was maintained in McCoy's 5A medium and terminated when visible clones appeared. Next, the cells were harvested and fixed with 4% paraformaldehyde for 15 minutes. The cells were then stained with crystal violet and dried at room temperature. Finally, the number of clones which contains more than 50 cells was counted under the microscope.

### 2.6. Tumor Invasion Assays

Caki-1 cells were trypsinized, resuspended in serum-free culture medium, and counted. The upper chamber membranes of Transwell were evenly covered with Matrigel (BD science, New Jersey, USA) and incubated for 1 h before the cells were seeded. After 24 hours of conventional culture, cells in the upper chamber were gently wiped off by a cotton swab. Each chamber was fixed with methanol, stained with crystal violet, washed with PBS, and dried at room temperature. Six fields were randomly selected to take pictures under a microscope.

### 2.7. Wound-Healing Assays

Cells were harvested and seeded on six-well plates with 5 × 10^5^ per well. To simulate a wound, the cell surface was scratched by a 10 *μ*l sterile plastic pipette tip with a straightedge when cells reached a confluent of 85% to 90%. Then, the cells were cultured in serum-free medium and incubated at 37°C. Overnight, wound images were collected under a microscope. The distance of the wound was measured and recorded, and the migration distance of cells in each group was calculated.

### 2.8. Cell Viability Assay

The proliferation of kidney cancer cells was determined by CCK-8 assays (Dojindo Molecular Technologies, Kumamoto, Japan). Generally, renal cancer cells were seeded into 96-well plates and incubated for 0 h, 24 h, 48 h, and 72 h. Then, the medium in each well was discarded and replaced by 100 *μ*l of serum-free medium mixed with 10 *μ*l of CCK-8 under dark condition. After incubated for 2 h, OD values at the absorbance of 470 nm were recorded.

### 2.9. Western Blot Analysis

Caki-1 cells were lysed by RIPA lysis buffer (Thermo Fisher Scientific Inc., MA, USA) mixed with protease and phosphatase inhibitors. Then, proteins were separated by SDS-PAGE and transferred to PVDF membranes. After transfer, membranes were put into 5% bovine serum albumin and sealed at 25°C for 2 hours. Next, membranes were washed thrice with TBST on a shaker for 5 minutes each time and incubated with TBST diluted primary JMJD4 antibody (AP1030a, Abcepta, San Diego, CA, USA) and anti-*β*-actin antibody (ab8227, Abcam, Cambridge, MA, USA) at 4°C. Overnight, membranes were washed by TBST and incubated with horseradish peroxidase- (HRP-) labeled secondary antibody (Jackson 1 : 2000) for 2 h at 25°C. Finally, membranes were reacted with electrochemical luminescence regents (Thermo Fisher Scientific Inc., MA, USA) and photographed in dark room.

### 2.10. Statistical Analysis

Clinical and RNA-seq data were organized and analyzed through R software 3.6.3 (Auckland, New Zealand) according to previous studies [14]. The box plots are used to analyze the expression level of JMJD4 between normal tissue and cancerous tissue among patients with various kinds of cancers including ccRCC. The relationship between JMJD4 expression and clinical characteristics of ccRCC patients was assessed by Wilcoxon signed rank test and Logistic regression. Univariate and multivariate Cox analyses were used to determine whether JMJD is an independent prognosis factor. Overall survival (OS), disease-specific survival (DSS), and progression-free interval (PFI) curves were plotted by the Kaplan–Meier method and compared with the log rank test. The diagnostic value of JMJD4 was assessed by plotting the received operating characteristic (ROC) curve and calculating the area under the curve. Significances were computed by Graphpad Prism 7.0 (Graphpad Software, Inc., CA, USA).

## 3. Results

### 3.1. Clinical Characteristics of ccRCC Patients

Totally, clinical data of 539 patients with ccRCC were downloaded from the official site of TCGA and analyzed. The characteristics of 539 patients are shown in Table 1. There are 353 male participants (65.5%) and 186 female participants (34.5%) with a median age of patients is 61. In terms of Fuhrman grade, 14 patients were grade I (2.6%), 235 patients were grade II (44.3%), 207 patients were grade III (39%), and 75 patients were grade IV (14.1%).

### 3.2. Expression of JMJD4 in ccRCC Tissues

**Figure 1:**
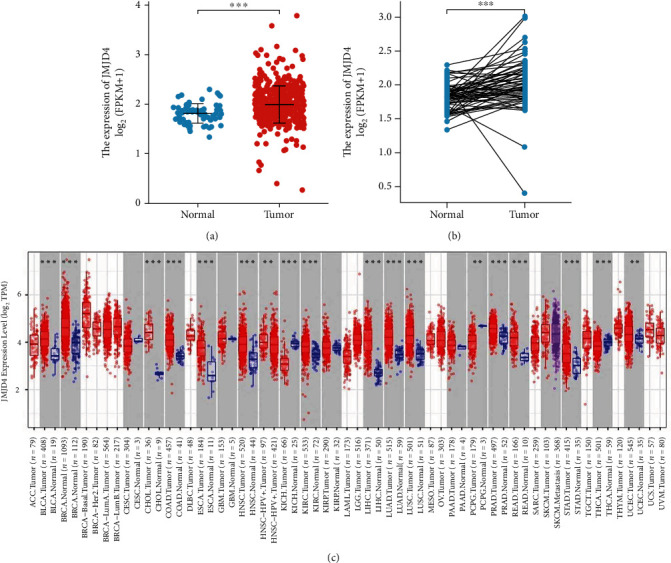
(a) JMJD4 expression in normal and tumor tissues. ∗∗∗ indicates a *p* value < 0.001. (b) JMJD4 expression in paired tissue. ∗∗∗ indicates a *p* value < 0.001. (c) JMJD4 expression in different types of human cancers in the TIMER database. ∗∗ indicates a *p* value < 0.01, and ∗∗∗ indicates a *p* value < 0.001.

JMJD4 was significantly higher in ccRCC tissues (*p* < 0.001) than in normal tissues both in paired and unpaired box plot analysis (Figures 1(a) and 1(b)). The expression of JMJD4 was also higher in bladder cancer, breast cancer, cholangiocarcinoma, colon cancer, renal cancer, lung cancer, etc., than in normal tissue (*p* < 0.01 or *p* < 0.001, Figure 1(c)).

### 3.3. Correlation of High Expression of JMJD4 with Clinical Characteristics

The relationships between JMJD4 and clinical characteristics of ccRCC patients are displayed in Table 2. JMJD4 showed significant relevance with M stage (*p* = 0.016), gender (*p* = 0.003), OS (0.018), DSS (0.007), and PFI (0.041). As shown in Table 3, Logistic regression analysis confirmed that high expression of JMJD4 was significantly associated with M stage (M1 vs. M0: OR = 1.876, 95%confidence interval (CI) = 1.151–3.102, and *p* = 0.013), pathological stage (stage III and stage IV vs. stage I and stage II, OR = 1.487 95%, CI = 1.048‐2.113, and *p* = 0.027), and gender (male vs. female, OR = 0.573, 95%CI = 0.399‐0.819, and *p* = 0.002).

### 3.4. High Expression of JMJD Predicts a Worse Clinical Outcome

**Figure 2:**
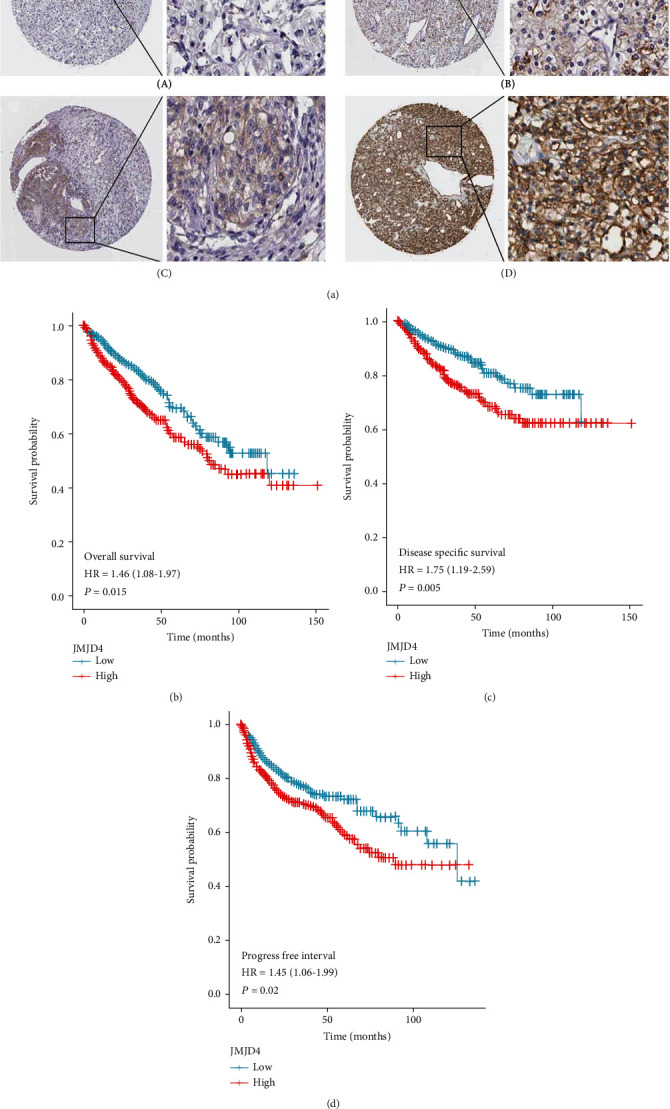
High expression level of JMJD4 portends a worse prognosis and outcome. (a) Online representative images of IHC staining of JMJD4 in cancerous tissue microarrays. A, minimum expression; B, low expression; C, moderate expression; and D, high expression. Scale bar = 100 *μ*m. (b–d) Kaplan–Meier curves of OS, DSS, and PFI.

**Figure 3:**
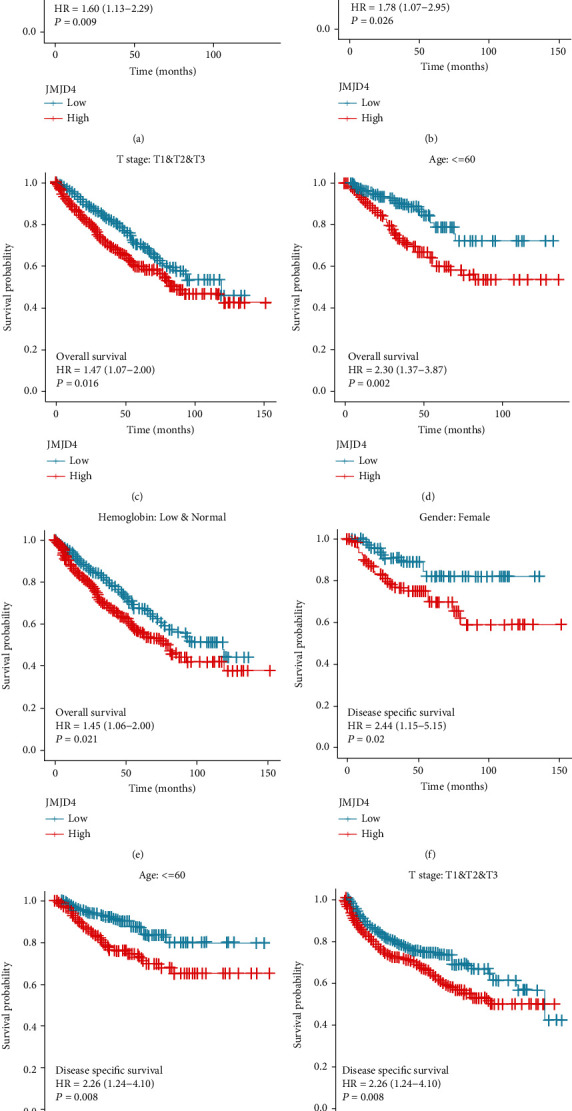
(a–e) Subgroup analysis of overall survival for patients in advanced Fuhrman grade, female patients, patients in T1 to T3 stage, patients age no greater than 60, and patients with low or normal hemoglobin. (f, g) Subgroup analysis of DSS for patients with ccRCC in female patients and patients age no greater than 60. (h) Subgroup analysis of PFI for patients with ccRCC in T1 to T3.

Online immunohistochemical staining images of JMJD4 in ccRCC patients are shown in Figure 2(a). We performed Kaplan–Meier survival analysis to assess the prognostic value of JMJD4, and results demonstrated that high JMJD4 expression was associated with poor OS (*p* = 0.015), DSS (0.005), and PFI (0.02) as shown in Figures 2(b)–2(d). Subgroup analysis by different clinical characteristics demonstrated that high JMJD4 expression was significantly associated with poor OS in patients with advance Fuhrman grade (*p* = 0.009), female patients (*p* = 0.026), patients from T1 to T3 stage (*p* = 0.016), patients no greater than 60 years old (*p* = 0.002), and patients with low or normal hemoglobin (*p* = 0.021) as shown in Figures 3(a)–3(e). High level of JMJD4 is also connected with poor PFI in patients from T1 to T3 stage (*p* = 0.021) and poor DSS in patients no greater than 60 years old (*p* = 0.008) and female patients (*p* = 0.02).

**Figure 4:**
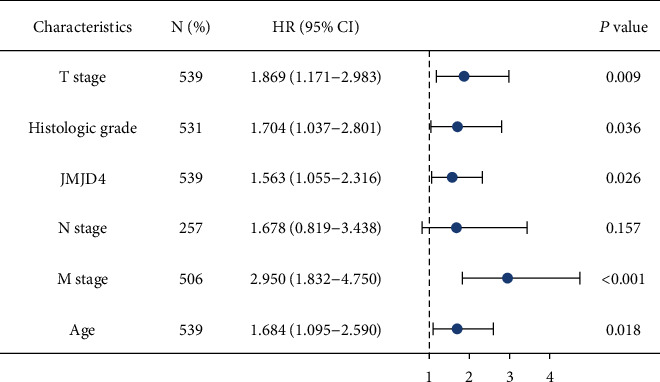
Forest plot of the multivariate Cox regression analysis in kidney cancer.

Univariate Cox regression analysis manifested that high JMJD4 expression was significantly correlated with poor OS (HR = 1.466, 95%CI = 1.084 − 1.983, and *p* = 0.013). Multivariate cox regression further validated that JMJD4 has independent prognostic value in patients with ccRCC (Table 4 and Figure 4, HR = 1.563, 95%CI = 1.055‐2.316, and *p* = 0.026).

### 3.5. Diagnostic Value of JMJD4 in ccRCC Patients

**Figure 5:**
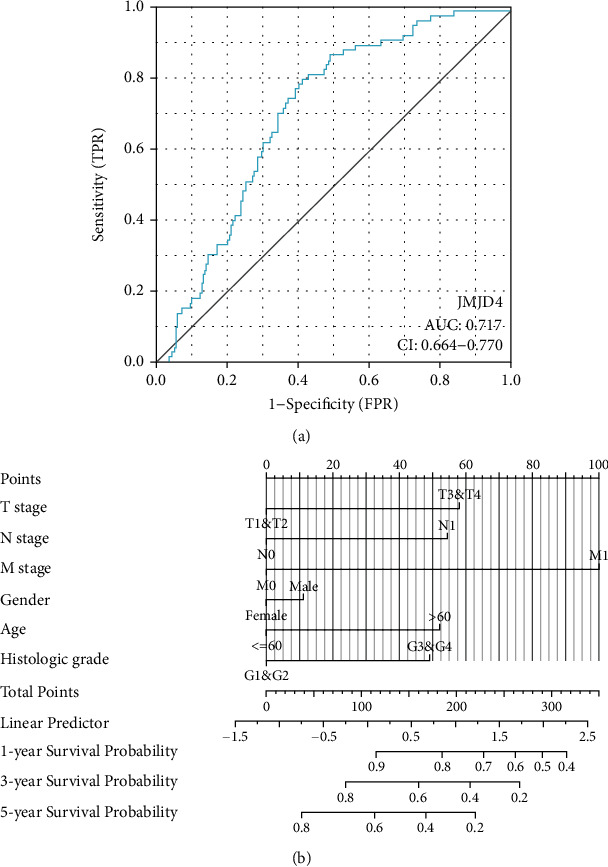
(a) ROC curve for JMJD4 in ccRCC patients. (b) Nomogram for predicting probability of patients with 1-, 3-, and 5-year overall survival.

The area under the ROC was 0.717, which indicates a high diagnostic value of JMJD for ccRCC patients (Figure 5(a)). The 1-, 3-, and 5-year survival probability of ccRCC patients are shown in Figure 5(b) by constructing a nomogram with the combination of JMJD4 and other clinical characteristics.

### 3.6. Genes Enriched in Different Signaling Pathways

**Figure 6:**
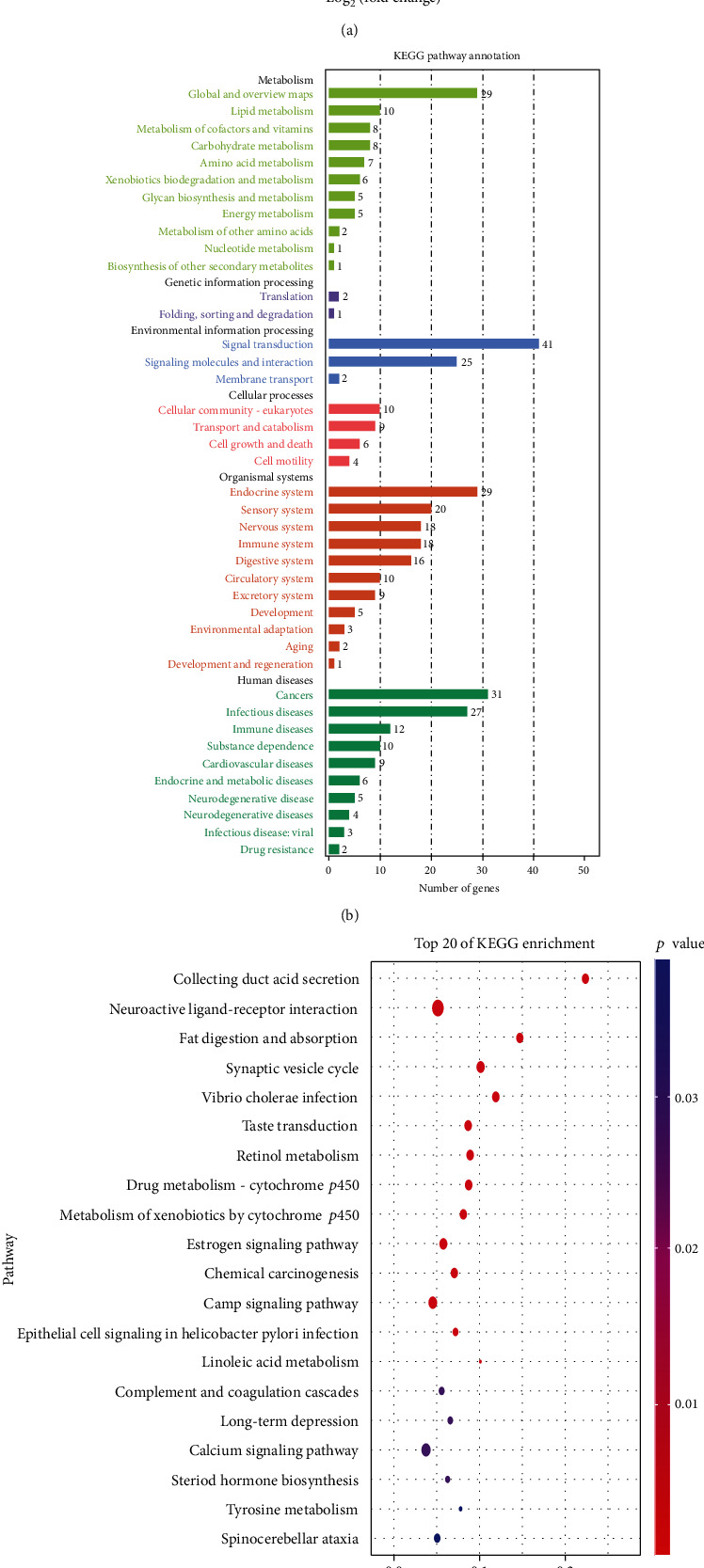
(a) Volcano map of the differentially expressed genes. (b) KEGG pathway analysis showed that genes mainly enriched in signal transduction and cancer disease. (c) Bubble diagram of top 20 most enriched genes in KEGG analysis.

**Figure 7:**
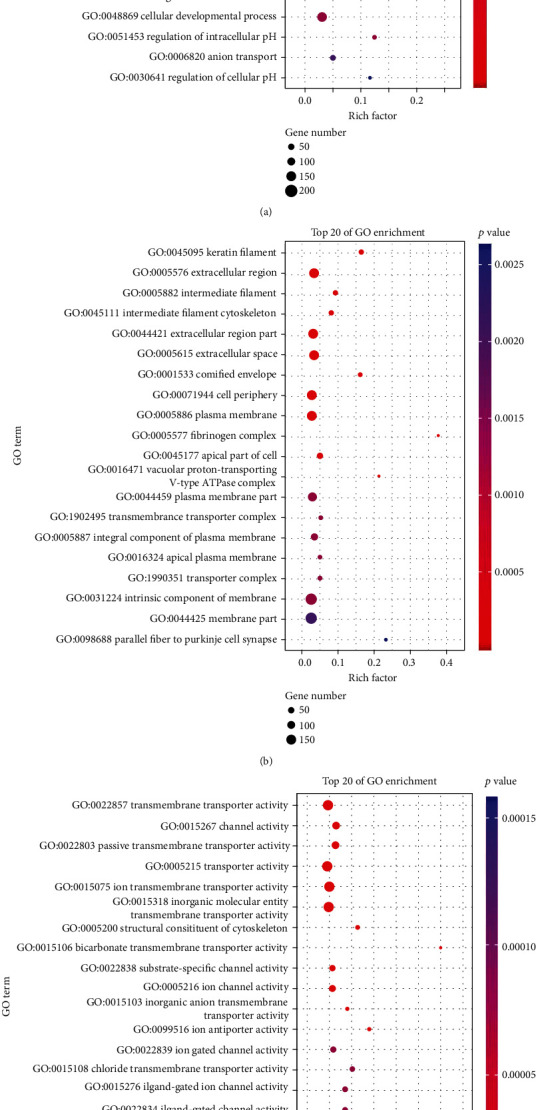
GO analysis. (a–c) Bubble diagram of top 20 most enriched genes in BP, CC, and MF analysis.

We analyzed the RNA-seq data from TCGA database. Volcano map of differentially expressed genes is shown in Figure 6(a), and more genes were downregulated. The result of KEGG pathway enrichment is shown in Figure 6(b). KEGG analysis has shown that three most enriched pathways were estrogen signaling pathway, Staphylococcus aureus infection, and neuroactive ligand-receptor pathway. There are 31 genes enriched in tumor-related pathways as shown in Figure 6(c), suggesting that JMJD4 is closely related to cancer development. GO analysis and KEGG pathway enrichment were performed to investigate genes correlated with JMJD4 associated with ccRCC. Pathways that meet *p* value < 0.05 and *q* value < 0.2 are considered to be statistically significant. In biological process (BP) of GO analysis, genes were mainly associated with cornification, keratinization, keratinocyte differentiation, epidermal cell differentiation, and skin development (Figure 7(a)). Significant pathways in cellular component (CC) included keratin filament, extracellular region, intermediate filament, intermediate filament cytoskeleton, and extracellular region part as shown in Figure 7(b). Most enriched genes in molecular function (MF) include transmembrane transporter activity, channel activity, passive transmembrane transporter activity, transporter activity, and ion transmembrane transporter activity (Figure 7(c)). Genes enriched in pathway mentioned above include DSG3, KRT4, KRT5, KRT6, KRT14, KRT15, KRT16, and IVL.

### 3.7. JMJD4 Promoted Renal Cancer Cell Proliferation, Invasion, and Colony Formation

**Figure 8:**
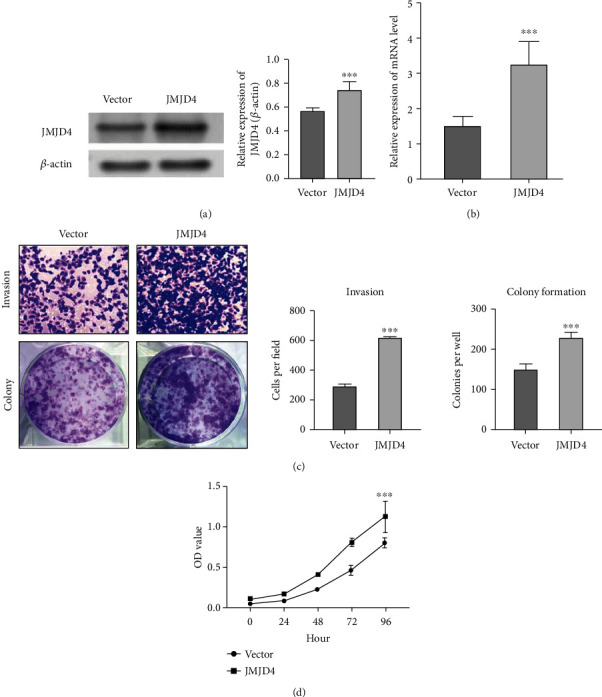
JMJD4 promoted cell proliferation, colony formation, and invasion in kidney cancer cells. (a, b) JMJD4 expression was validated by western blot and qRT-PCR assay. ∗∗∗ indicates a *p* value < 0.001. (c) Overexpressed JMJD4 promotes the invasion and colony formation capability of Caki-1 cells. ∗∗∗ indicates a *p* value < 0.001. (d) CCK-8 proliferation curve of Caki-1 cells. ∗∗∗ indicates a *p* value < 0.001.

To exploit the role of JMJD4 in renal cell proliferation and development, plasmid containing pcDNA-3.1(+)-JMJD4 was transfected into the Caki-1 cell line. The Transwell invasion assay and colony formation assay were then used to unravel the effect of JMJD4 on cell invasion and colony formation, and the results showed that JMJD4 significantly facilitated the invasion and colony formation ability of Caki-1 cells (*p* < 0.001, Figure 8(a)). The efficiency of overexpression was verified by qRT-PCR and western blot (Figures 8(b) and 8(c)). As a result, western blot analysis and qRT-PCR showed that the expression of JMJD4 was significantly upregulated after transfection of pcDNA-3.1(+)-JMJD4. To investigate the effect that JMJD4 exerts on renal cell proliferation, we performed CCK-8 assays. The results showed that overexpression of JMJD4 in Caki-1 cells promoted the renal cell growth (*p* < 0.001, Figure 8(d)).

## 4. Discussion

JumonjiC (JmjC) domain-containing histone demethylase (JMJD) is a protein family that plays an important role in cancer growth and development by regulating gene transcription and chromatin structure. Among the members of family, JMJD6 was reported to be tumorigenic factor which involves in various kinds of cancers such as prostate cancer [15], breast cancer [16, 17], melanoma [18], colon cancer [19], and lung cancer [20]. JMJD4 is a paralog of JMJD6 which shares 34% sequence with JMJD6 [21]. Knockout of the JMJD6 gene in mice will significantly reduce the proliferation of NIH3T fibroblasts, while knocking out JMJD4 will cause the same effect by RNA interference [22]. Study has confirmed that JMJD4-knockout embryos are born at a normal Mendelian ratio which proved that JMJD4 is unessential in embryogenesis [23]. Currently, only one study has reported that JMJD4 expression could be a prognostic indicator for patients with colon cancer [24]. The interaction between JMJD4 and other tumors still needs more research to reveal. Therefore, we investigate the relationship between JMJD4 and renal cancer.

In this study, we first use bioinformatics analysis to assess the role of JMJD4 in ccRCC from online public database such as TCGA and TIMER. The results of box plot analysis demonstrate that high expression of JMJD4 is a common feature of multiple human cancers, including ccRCC. And Logistic regression analysis indicated that high JMJD4 is closely related to M stage, gender, and prognosis in ccRCC patients. Then, to evaluate the prognostic value of JMJD4 in ccRCC patients, we performed Kaplan–Meier survival analysis, and results showed that patients with high JMJD4 expression had poor OS, DSS, and PFI. Moreover, Cox regression analysis confirmed that high JMJD4 expression has independent prognostic value for OS in ccRCC patients. Other clinicopathological characteristics are also related to the poor OS of ccRCC, including advanced Fuhrman grade, M stage, T stage, and age, as shown in Figure 4. Next, we performed ROC analysis and compute the AUC. The results suggest that high JMJD4 expression has certain diagnostic value in ccRCC patients (Figure 5(a)). Besides, to help practitioners predict the risk of individual death and optimize treatment options, we also constructed a prognostic nomogram (Figure 5(b)).

To further evaluate the mechanism of high expression of JMJD4 in promoting the progression of renal cancer, we performed GO and KEGG analysis. We found that the high JMJD4 expression was associated with collecting duct acid secretion, neuroactive ligand-receptor interaction, and fat digestion and absorption pathway in KEGG analysis. GO analysis shows that keratinization, keratinocyte differentiation, and epidermal cell differentiation were most enriched genes in BP. Skin development extracellular region, intermediate filament, and intermediate filament cytoskeleton were most enriched pathways in CC, while transmembrane transporter activity, channel activity, and passive transmembrane transporter activity were most enriched pathways in MF. The main genes enriched in these pathways include DSG3, KRT5, KRT6, and KRT14. DSG3 was reported to promote the growth and invasion of cancer cells [25]. KRT5 deficiency can prevent the migration of ovarian cancer cells, which prompts that it may act as an oncogene [26]. KRT6 is associated with notch1 signaling and contributes the progression of renal cancer [27]. KRT14 was proved to be a key regulator in metastasis of breast cancer [28]. The above data shows that the genes related to JMJD4 are mainly cancer-promoting genes, indicating that JMJD4 may promote the invasion and progression of cancer. Furthermore, to clarify the effect of JMJD4 on kidney cancer, we transfected the pcDNA-3.1(+)-JMJD4 plasmid into Caki-1 cells and verified the overexpression by qRT-PCR and western blot (Figures 8(a) and 8(b)). Then, we performed Transwell invasion experiments and plate colony formation experiments. The results showed that JMJD4 overexpression significantly promoted the invasiveness and colony formation ability of Caki-1 cells (Figures 8(c), *p* < 0.001). We also conducted the CCK-8 experiment, and the results demonstrate that JMJD4 can significantly promote the proliferation of Caki-1 cells (Figures 8(d), *p* < 0.001). The above *in vitro* data prove that JMJD4 acts as a cancer-promoting gene in renal cancer cells. One limitation of our research is that we did not conduct *in vivo* experiments for verification. Subsequent experiments can be performed on animal experiments to verify the cancer-promoting effect of JMJD4 *in vivo.*

In summary, our study is the first research that focuses on the role of JMJD4 played in ccRCC. We validated the predictive value and diagnostic value of JMJD4 in ccRCC patients, as well as the effect of JMJD4 on promoting the invasion and proliferation of ccRCC cells. Our results showed that high expression of JMJD can be a potential prognostic marker for ccRCC patients.

## Figures and Tables

**Table 1:** Clinical characteristics of ccRCC patients.

Characteristic	Levels	Overall
*n*		539

T stage, *n* (%)	T1	278 (51.6%)
	T2	71 (13.2%)
	T3	179 (33.2%)
	T4	11 (2%)

N stage, *n* (%)	N0	241 (93.8%)
	N1	16 (6.2%)

M stage, *n* (%)	M0	428 (84.6%)
	M1	78 (15.4%)

Pathologic stage, *n* (%)	Stage I	272 (50.7%)
	Stage II	59 (11%)
	Stage III	123 (22.9%)
	Stage IV	82 (15.3%)

Primary therapy outcome, *n* (%)	PD	11 (7.5%)
	SD	6 (4.1%)
	PR	2 (1.4%)
	CR	128 (87.1%)

Gender, *n* (%)	Female	186 (34.5%)
	Male	353 (65.5%)

Race, *n* (%)	Asian	8 (1.5%)
	Black or African American	57 (10.7%)
	White	467 (87.8%)

Age, *n* (%)	≤60	269 (49.9%)
	>60	270 (50.1%)

Histologic grade, *n* (%)	G1	14 (2.6%)
	G2	235 (44.3%)
	G3	207 (39%)
	G4	75 (14.1%)

Age, median (IQR)		61 (52, 70)

**Table 2:** Correlation between the expression of JMJD4 and clinical characteristics.

Characteristic	Low expression of JMJD4	High expression of JMJD4	*p*
*n*	269	270	
T stage, *n* (%)			0.369
T1	147 (27.3%)	131 (24.3%)	
T2	37 (6.9%)	34 (6.3%)	
T3	80 (14.8%)	99 (18.4%)	
T4	5 (0.9%)	6 (1.1%)	
N stage, *n* (%)			0.224
N0	136 (52.9%)	105 (40.9%)	
N1	6 (2.3%)	10 (3.9%)	
M stage, *n* (%)			0.016
M0	231 (45.7%)	197 (38.9%)	
M1	30 (5.9%)	48 (9.5%)	
Pathologic stage, *n* (%)			0.083
Stage I	144 (26.9%)	128 (23.9%)	
Stage II	34 (6.3%)	25 (4.7%)	
Stage III	58 (10.8%)	65 (12.1%)	
Stage IV	32 (6%)	50 (9.3%)	
Primary therapy outcome, *n* (%)			0.762
PD	4 (2.7%)	7 (4.8%)	
SD	4 (2.7%)	2 (1.4%)	
PR	1 (0.7%)	1 (0.7%)	
CR	63 (42.9%)	65 (44.2%)	
Gender, *n* (%)			0.003
Female	76 (14.1%)	110 (20.4%)	
Male	193 (35.8%)	160 (29.7%)	
Race, *n* (%)			0.091
Asian	5 (0.9%)	3 (0.6%)	
Black or African American	21 (3.9%)	36 (6.8%)	
White	240 (45.1%)	227 (42.7%)	
Age, *n* (%)			0.111
≤60	144 (26.7%)	125 (23.2%)	
>60	125 (23.2%)	145 (26.9%)	
Histologic grade, *n* (%)			0.073
G1	6 (1.1%)	8 (1.5%)	
G2	124 (23.4%)	111 (20.9%)	
G3	106 (20%)	101 (19%)	
G4	27 (5.1%)	48 (9%)	
OS event, *n* (%)			0.018
Alive	196 (36.4%)	170 (31.5%)	
Dead	73 (13.5%)	100 (18.6%)	
DSS event, *n* (%)			0.007
Alive	223 (42.2%)	197 (37.3%)	
Dead	41 (7.8%)	67 (12.7%)	
PFI event, *n* (%)			0.041
Alive	200 (37.1%)	178 (33%)	
Dead	69 (12.8%)	92 (17.1%)	
Age, mean ± SD	59.87 ± 12.11	61.39 ± 12.05	0.145

**Table 3:** Logistic regression analysis of the association between JMJD4 expression and clinical characteristics.

Characteristics	Total (*N*)	Odds ratio(OR)	*p* value
T stage (T3 & T4 vs. T1 & T2)	539	1.378 (0.967-1.967)	0.077
N stage (N1 vs. N0)	257	2.159 (0.776-6.522)	0.148
M stage (M1 vs. M0)	506	1.876 (1.151-3.102)	0.013
Gender (male vs. female)	539	0.573 (0.399-0.819)	0.002
Age (>60 vs. ≤60)	539	1.336 (0.953-1.876)	0.093
Histologic grade (G3 & G4 vs. G1 & G2)	531	1.224 (0.870-1.723)	0.246
Race (Black or African American & White vs. Asian)	532	1.679 (0.408-8.255)	0.481
Primary therapy outcome (PR & CR vs. PD & SD)	147	0.917 (0.325-2.541)	0.866
Pathologic stage (stage III & stage IV vs. stage I & stage II)	536	1.487 (1.048-2.113)	0.027

**Table 4:** Cox regression analyses of clinical characteristics associated with overall survival.

Characteristics	Total (*N*)	Univariate analysis		Multivariate analysis	
		Hazard ratio (95% CI)	*p* value	Hazard ratio (95% CI)	*p* value
T stage	539				
T1 & T2	349	Reference			
T3 & T4	190	3.228 (2.382-4.374)	<0.001	1.869 (1.171-2.983)	0.009
Histologic grade	531				
G1 & G2	249	Reference			
G3 & G4	282	2.702 (1.918-3.807)	<0.001	1.704 (1.037-2.801)	0.036
JMJD4	539				
Low	270	Reference			
High	269	1.466 (1.084-1.983)	0.013	1.563 (1.055-2.316)	0.026
N stage	257				
N0	241	Reference			
N1	16	3.453 (1.832-6.508)	<0.001	1.678 (0.819-3.438)	0.157
M stage	506				
M0	428	Reference			
M1	78	4.389 (3.212-5.999)	<0.001	2.950 (1.832-4.750)	<0.001
Age	539				
≤60	269	Reference			
>60	270	1.765 (1.298-2.398)	<0.001	1.684 (1.095-2.590)	0.018
Gender	539				
Female	186	Reference			
Male	353	0.930 (0.682-1.268)	0.648		

## Data Availability

The datasets generated during the current study are available from the corresponding author on reasonable request.

## Supplementary Materials

Supplementary 1 Supplemental material 1: details of the enriched genes in KEGG analysis.

Supplementary 2 Supplemental material 2: details of the enriched genes in biological process of GO analysis.

Supplementary 3 Supplemental material 3: details of the enriched genes in molecular function of GO analysis.

Supplementary 4 Supplemental material 4: details of the enriched genes in cellular component of GO analysis.
